# Signet-ring cell carcinoma of the breast: a case report

**DOI:** 10.1186/1477-7819-11-183

**Published:** 2013-08-09

**Authors:** Xing Li, Yan-fen Feng, Wei-dong Wei, Peng Liu, Ze-ming Xie, Jin Wang, Xiao-ming Xie

**Affiliations:** 1 State Key Laboratory of Oncology in South China, Sun Yat-sen University Cancer Center, Guangzhou, Guangdong 510060, China; 2 Department of Breast Oncology, Sun Yat-sen University Cancer Center, Guangzhou, Guangdong 510060, China; 3 Department of Pathology, Sun Yat-sen University Cancer Center, Guangzhou, Guangdong 510060, China

**Keywords:** Signet-ring cell carcinoma breast cancer

## Abstract

Signet-ring cell carcinoma (SRCC) can arise from virtually all organs. However, primary SRCC of the breast is very rare. Until 2003, SRCC was placed under ‘mucin-producing carcinomas’ and separated from other carcinomas by the World Health Organization (WHO). To date, only a few cases have been reported. A case of a 46-year-old woman with primary SRCC of the breast is presented in this report. The patient underwent a right modified radical mastectomy with axillary lymph node dissection. Characteristic features and differential diagnosis of this tumor are discussed in the light of pertinent literature.

## Background

Signet-ring cell carcinoma (SRCC) is a unique subtype of mucin-producing adenocarcinoma that can arise from the stomach, breast, colon, lung and prostate [1]. Primary SRCC of the breast is a very rare tumor, which shows a significant number of tumor cells with intracellular mucin accumulation [2,3]. In 1941, Saphir first described this type of tumor as a variety of mucinous carcinoma [4]. In 1976, Steinbrecher and Silverberg reported five cases of rare carcinoma of the breast characterized by the presence of numerous cells containing intracellular mucin, without large amounts of extracellular mucins seen in colloid carcinoma of the breast [5]. Hull *et al*. found 24 cases of SRCC, which represented 4.5% of 535 cases of surgically treated carcinomas of the breast in their database; most cases were associated with the ductal carcinoma and lobular carcinoma, and only four cases were pure SRCC [2]. Until 2003, SRCC was placed under ‘mucin-producing carcinomas’, and separated from both infiltrating ductal and lobular carcinomas by the World Health Organization (WHO) [6].Therefore, the frequency of this tumor was difficult to evaluate because it was not a distinct type.

In this case report, we present a case of pure SRCC of the breast. The characteristic clinical, morphological and immunohistochemical features, and differential diagnosis of this tumor are discussed.

## Case presentation

A 46-year-old woman presented to Sun Yat-sen University Cancer Center, Guangzhou, China, with a lump in the right breast discovered by self-examination one month ago. The patient had no previous breast problems or family history of breast cancer. Physical examination revealed a firm, 10 mm × 5 mm mass in the upper outer quadrant of the right breast, without evidence of axillary or supraclavicular lymphadenopathy. There was no tethering of the skin or *peaud’orange*. The contralateral breast and axilla were normal, and the tested blood parameters, chest X-ray, electrocardiography (ECG) and heart functions were also normal.

Ultrasonography of the right breast showed a hypoechoic area measuring approximately 10 mm × 7 mm in the upper outer quadrant, demonstrating a circumscribed complex echoic mass with posterior enhancement, uneven density and abundant vessels. A preoperative biopsy of the breast was planned under the guidance of ultrasound. Mammography revealed heterogeneously dense breasts, without any evidence of mass lesions, architectural distortion or microcalcifications.

Fine-needle aspiration cytology (FNAC) of the breast tissue demonstrated that the tumor was SRCC, but it was needed to distinguish whether it was derived from metastasesto the breast from extramammary sites.

Gastrointestinal endoscopy was normal. Computerized tomography (CT) scan of the neck, chest and upper abdomen showed several enlarged lymph nodes in the neck, but the thorax and abdomen were normal. Gastrointestinal ultrasonography also showed no abnormality.

The patient underwent a right modified mastectomy with axillary lymph node dissection, and received the first course of chemotherapy 15 days later.

### Histological findings

**Figure 1:**
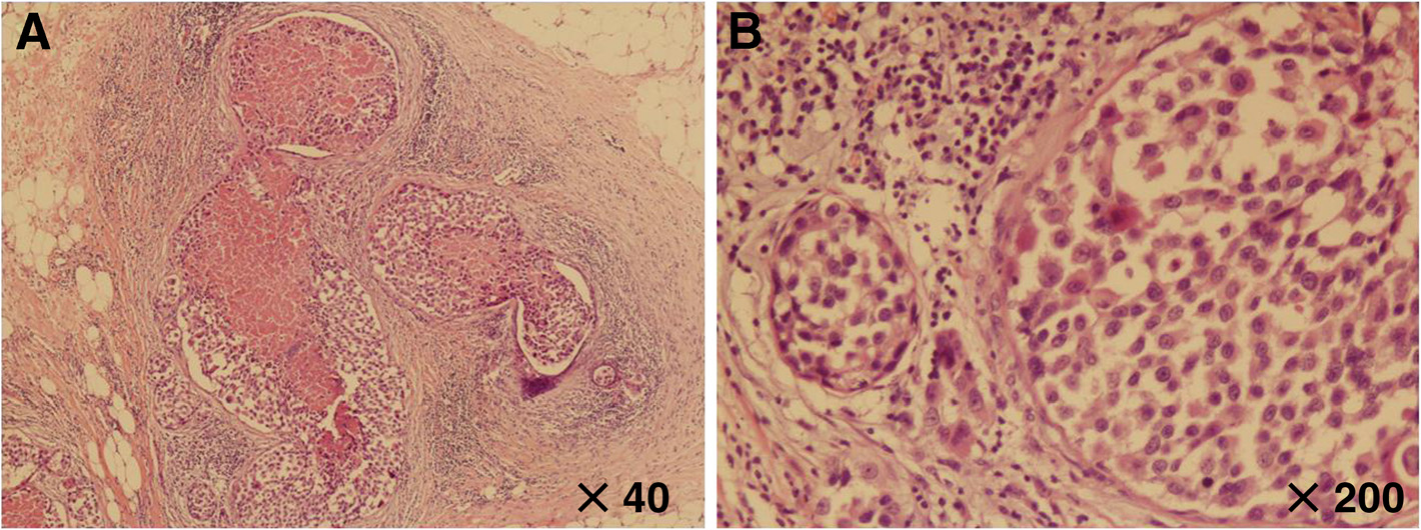
**Histological findings of case study.** The carcinoma showed invasive carcinoma, and partial presence of the *in situ* component of carcinoma. **(A)** H&E stain, magnification × 40; **(B)** H&E stain, magnification × 200.

**Figure 2:**
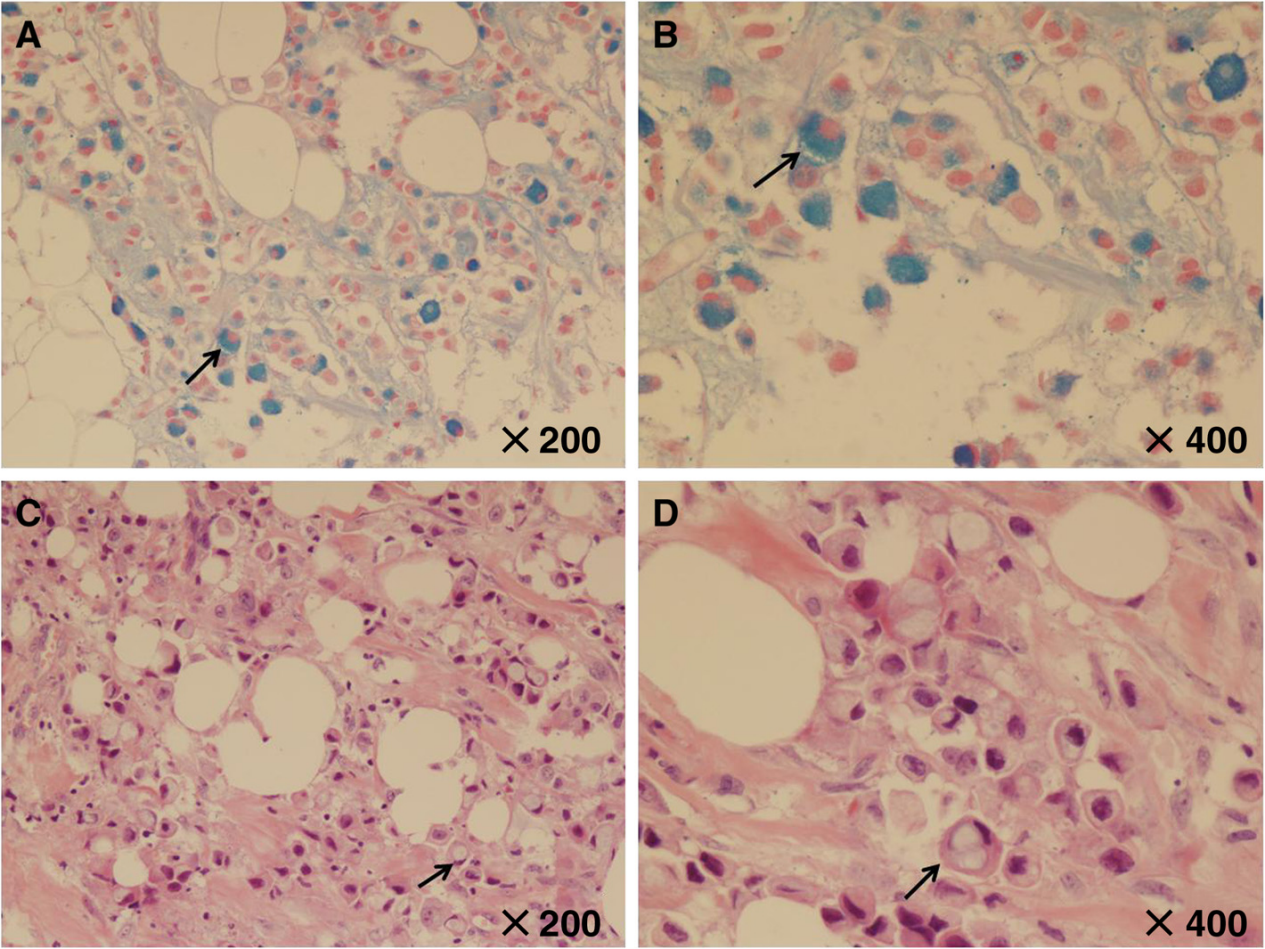
**Histological findings of case study.** The carcinoma component comprised of intracytoplasmic mucin with **(A, B)** Alcianblue staining and **(C, D)** signet-ring tumor cells.

Gross pathologic examination of the right breast specimen revealed a tumor, which measured 3.5 cm × 2.5 cm × 1.5 cm in diameter. Microscopically, the right surgically resected mass showed invasive carcinoma and partial presence of the *in situ* component of carcinoma (Figure 1). The histological examination of the neoplastic cells showed that they were small and round, scattered or funicular distribution, and with large intracytoplasmic mucin compressing the nuclei toward one pole of the cell (Figure 2). The majority (>50%) of tumor cells showed features of signet-ring cells, with high-grade nuclear atypia, moderate mitotic activity and high proliferation index (Ki-67 >40%). The diagnosis of the tumor was established as SRCC. There were 14 metastases found in 21 lymph nodes, with tumor emboli in vessel.

### Immunohistochemical findings

**Figure 3:**
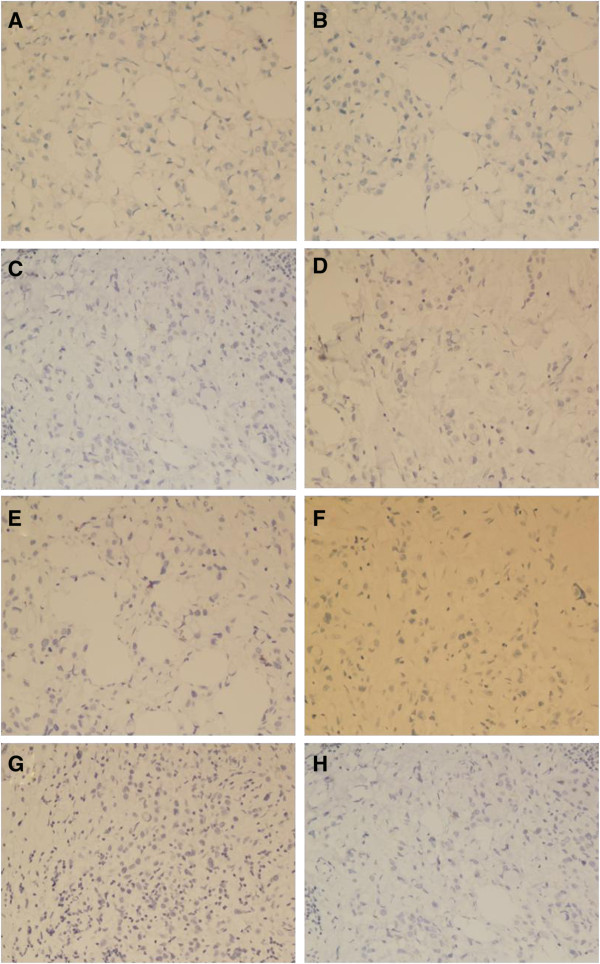
**Immunohistochemical findings of the tumor.** The signet-ring cell carcinoma (SRCC) component was negative for **(A)** ER, **(B)** PR, **(C)** GCDFP-15, **(D)** mammaglobin, **(E)** Hep Par1, **(F)** TTF-1, **(G)** MUC2 and **(H)** CK20; magnification × 200. CK20, cytokeratin 20; ER, estrogen receptor; GCDFP-15, gross cystic disease fluid protein-15; Hep Par1, hepatocyte paraffin 1; MUC2, mucin 2 glycoprotein; PR, progesterone receptor; SRCC, signet-ring cell carcinoma; TTF-1, thyroid transcription factor-1.

**Figure 4:**
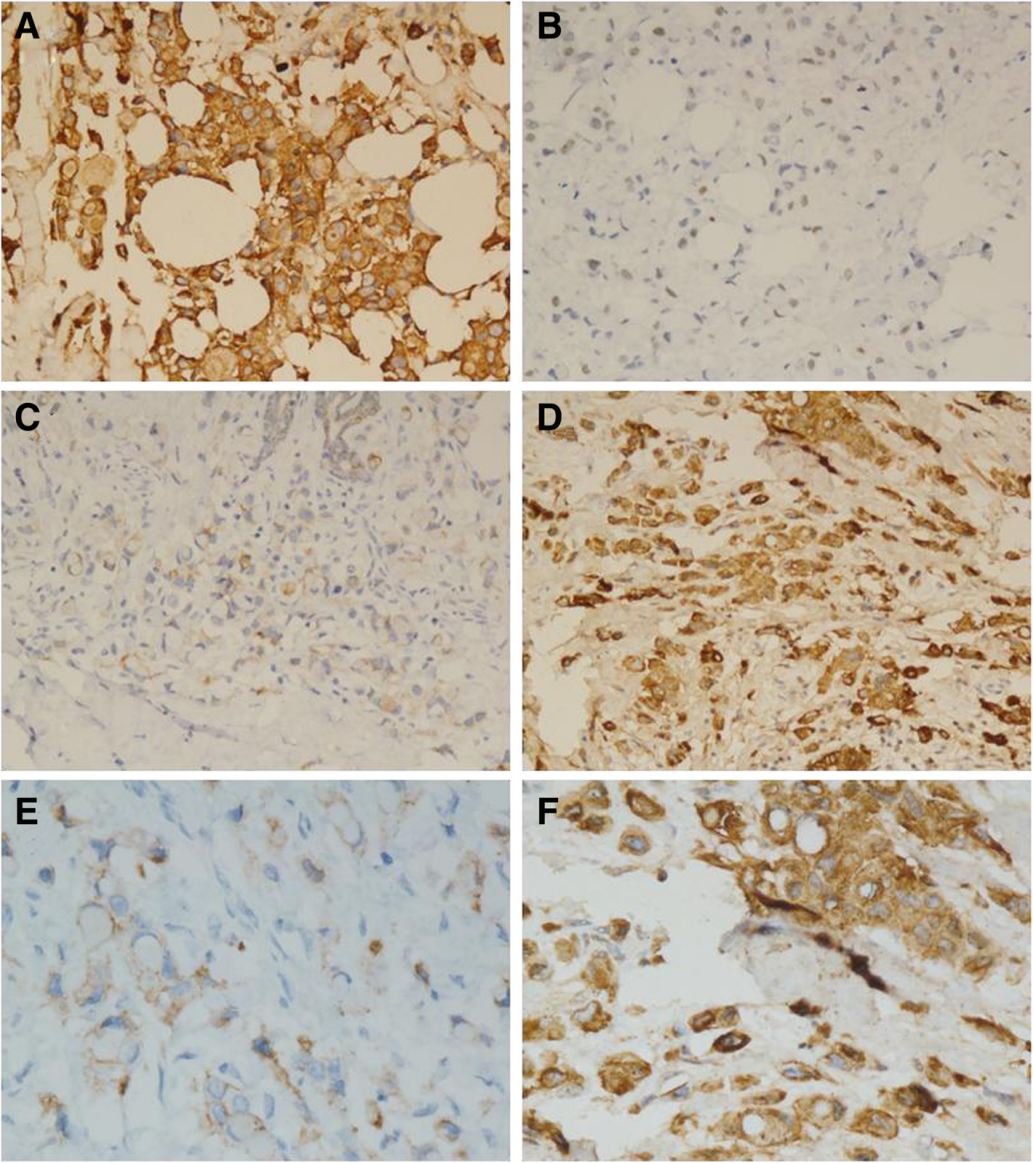
**Immunohistochemical findings of the tumor.** The signet-ring cell carcinoma (SRCC) component was positive for **(A)** MUC1, **(B)** CDX2, **(C)** E-ca and **(D)** CK7; magnification × 200; and furthermore, **(E)** E-ca and **(F)** CK7; magnification × 400. CDX2, caudal type homeobox 2; CK7, cytokeratin 7; E-ca, E-cadherin; MUC1, mucin 1 glycoprotein; SRCC, signet-ring cell carcinoma.

Immunohistochemical examination of the neoplastic cells demonstrated that part of the epithelial components expressed cytokeratin. Tumor cells showed a negative reaction for estrogen receptor (ER), progesterone receptor(PR), gross cystic disease fluid protein-15 (GCDFP-15), mammaglobin, hepatocyte paraffin 1 (Hep Par1), thyroid transcription factor-1 (TTF-1), mucin 2 glycoprotein (MUC2) and cytokeratin 20 (CK20) (Figure 3); and a positive reaction for mucin 1 glycoprotein (MUC1), caudal type homeobox 2 (CDX2), E-cadherin (E-ca) and cytokeratin 7 (CK7), but weak expression focal for E-ca (Figure 4). Table 1 presents a summary of antibodies used in the assessment of the lesion.

**Table 1:** Summary of antibodies used in the assessment of the lesion

**Antibody**	**Company**	**Clone**	**Dilution**
ER	Ventana	SP1	1:50
PR	Ventana	IE2	1:150
GCDFP-15	Dako	23A3	1:50
Mammaglobin	Zeta Corporation	304-1A5	1:120
MUC1	Cell Marque	Ma695	1:120
MUC2	Zeta Corporation	Ccp58	1:120
CK7	Invitrogen	OV-TL12/30	1:150
CK20	Epitomics	EP23	1:150
CDX2	Epitomics	EP25	1:200
E-ca	Epitomics	EP6	1:150
Hep Par 1	Zeta Corporation	OCH1E5	1:150
TTF-1	LeicaMicrosystems	SPT24	1:180

Cell Marque, Rocklin, CA, USA; Dako, Carpinteria, CA, USA; Epitomics, Burlingame, CA, USA; Invitrogen, Carlsbad, CA, USA; Leica Microsystems,Wetzlar, Germany; Ventana, Oro Valley, AZ, USA; Zeta Corporation, Sierra Madre, CA, USA.CDX2, caudal type homeobox 2; CK7, cytokeratin 7; CK20, cytokeratin 20; E-ca, E-cadherin; ER, estrogen receptor; GCDFP-15, gross cystic disease fluid protein-15; Hep Par1, hepatocyte paraffin 1; MUC1, mucin 1 glycoprotein; MUC2, mucin 2 glycoprotein; PR, progesterone receptor; TTF-1, thyroid transcription factor-1.

## Discussion

Until now, only a few cases of SRCC of the breast have been reported in the English literature, and the prevalence of signet-ring features has ranged from 2 to 4.5% of total breast cancers [2,7,8].

SRCC of the breast can be divided into primary and metastatic tumors. A variety of immunohistochemical markers have been applied to distinguish SRCC from different organs. SRCCs of the breast are generally immunohistochemically positive for GCDFP-15, whereas SRCCs of the gastrointestinal tract are negative [9]. Furthermore, adenocarcinomas of the breast, stomach and colon show different CK7 and CK20 expression patterns [10-12]. ER is very often positive in primary SRCC of the breast, but commonly negative in gastric and colonic signet-ring cells. While primary SRCC of the breast is typically positive for CK7 but negative for CK20, the gastrointestinal SRCCs are commonly positive for CK20 but usually negative for CK7. In combination with ER staining, CK7 and CK20 expression patterns can be used to distinguish gastrointestinal SRCC from SRCC of the breast. Although ER is usually expressed in carcinoma of the breast, approximately 20% of SRCCs of the breast can be negative for ER [12]. In this case report, neoplastic cells showed negative reaction for ER, PR, CK20 and GCDFP-15, and positive reaction for CK7.

In recent years, new antibodies have been found useful in the differential diagnosis. Adenocarcinomas of the breast usually express MUC1 but not MUC2, whereas gastrointestinal adenocarcinomas frequently express MUC2 but less frequently express MUC1 [13,14]. The combination of immunomarkers can substantially increase the sensitivity and specificity for diagnosing SRCCs of these organs. Chu *et al*. [1] found that the use of a SRCC of the breast can be distinguished from gastrointestinal SRCC, if ER andMUC1 are used as markers for SRCC of the breast, and MUC2 and CDX2 can be used as markers for gastric and colon SRCCs. To distinguish SRCCs of gastric versus colonic origin, Hep Par1 and CDX2 strongly favor a gastric primary site, whereas Hep Par1 negativity and MUC2 positivity strongly favor a colonic primary site. E-ca is less useful and lacks sensitivity. In this report, tumor cells showed positive reaction for E-ca, CDX2 and MUC1, and negative reaction for MUC2, Hep Par1 and TTF-1. The carcinoma of this report comprised of intracytoplasmic mucin and signet-ring tumor cells, with a lack of typical features on immunohistochemical examination, but no sign of tumor in any other organs by radiology imaging. The immunophenotype of this case is highly unusual and the presence of a coexisting *in situ* ductal component strongly favors a primary breast site.

Regardless of the tissue origin, SRCCs frequently metastasize to regional lymph nodes, peritoneal surfaces, ovaries and lungs [1]. In pure SRCC of the breast, the lesion is more aggressive than mucinous carcinoma, invasive ductal carcinoma of no special type and classic invasive lobular carcinoma [3]. In this case report, there were 14 metastases found in 21 lymph nodes, with tumor emboli in vessel. The presence of 10% or more of signet-ring cells has been reported to be a poor individual prognostic factor in stage I infiltrating lobular carcinomas [15]. Consequently, it is important to distinguish primary and metastatic tumors because of their significant difference in therapy and prognosis. Immunohistochemistry may be particularly helpful in differentiating the tumors [2,3,16-19].

Treatment and prognosis of SRCC of the breast has been reported less frequently in the literature on account of its rarity. However, Eltorky *et al*. [20] reported that both the pathologist and the clinician should be aware of the prognostic influence of hormone receptor studies in the management of SRCC of the breast.

## Conclusions

Primary SRCC of the breast is a very rare malignant tumor and must be distinguished from metastases of SRCCs to the breast. The prognosis of this tumor is usually poor but early detection may provide a good result. It is important to differentiate this type of tumor according to the pathological and clinical characteristics.

## Consent

Written informed consent was obtained from the patient for publication of this case report and any accompanying images. A copy of the written consent is available for review by the Editor-in-Chief of this journal.

## Abbreviations

CDX2: caudal type homeobox 2; CK7: cytokeratin 7; CK20: cytokeratin 20; CT: computerized tomography; E-ca: E-cadherin; ECG: electrocardiography; ER: estrogen receptor; FNAC: fine-needle aspiration cytology; GCDFP-15: gross cystic disease fluid protein-15; H&E: hematoxylin and eosin; Hep Par1: hepatocyte paraffin 1; MUC1: mucin 1 glycoprotein; MUC2: mucin 2 glycoprotein; PR: progesterone receptor; SRCC: signet-ring cell carcinoma; TTF-1: thyroid transcription factor-1; WHO: World Health Organization.

## Competing interests

The authors declare that they have no competing interests.

## Authors’ contributions

XL and XMX drafted the article; YFF provided the pathology figure and edited the pathology component of the paper; WWD, PL, ZMX and JW supervised the writing of the paper. All authors read and approved the final manuscript.
